# Prenatal transposition of great arteries diagnosis and management: a Chinese single-center study

**DOI:** 10.3389/fcvm.2024.1341005

**Published:** 2024-03-06

**Authors:** Jie Guo, Wen Ling, Tingting Dang, Shan Guo, Hong Ma, Qiong Huang, Liqin Zeng, Zongjie Weng, Qiumei Wu

**Affiliations:** ^1^ Department of Medical Ultrasonics, Fujian Maternity and Child Health Hospital, College of Clinical Medicine for Obstetrics & Gynecology and Pediatrics, Fujian Medical University, Fuzhou, China; ^2^ Department of Pathology, Fujian Maternity and Child Health Hospital, College of Clinical Medicine for Obstetrics & Gynecology and Pediatrics, Fujian Medical University, Fuzhou, China

**Keywords:** early pregnancy, transposition of the great arteries, prenatal ultrasound, follow-up management, outcome

## Abstract

**Objective:**

This study aimed to assess the diagnostic value of prenatal echocardiography for identifying transposition of the great arteries (TGA) during pregnancy and evaluating the associated outcomes.

**Methods:**

We conducted a retrospective analysis of 121 prenatally diagnosed patients with TGA at our hospital between January 2012 and September 2022. This analysis included prenatal ultrasound, prenatal screening, clinical management and follow-up procedures.

**Results:**

Among the 103 fetuses considered in the study, 90 (87.4%) were diagnosed with complete transposition of the great arteries (D-TGA), while 13 (12.6%) exhibited corrected transposition of the great arteries (CC-TGA). Diagnoses were distributed across the trimester, with 8 D-TGA and 2 CC-TGA patients identified in the first trimester, 68 D-TGA patients and 9 CC-TGA patients in the second trimester, and 14 D-TGA and 2 CC-TGA patients referred for diagnosis in the third trimester. Induction of labour was pursued for 76 D-TGA patients (84.4%) and 11 CC-TGA patients (84.6%), and 14 D-TGA patients (15.6%) and 2 CC-TGA patients (15.4%) continued pregnancy until delivery. Among the D-TGA patients, 9 fetuses (10.0%) underwent surgery, two of which were inadvertent fatality, while the remaining seven experienced positive outcomes. Additionally, seven TGA patients received palliative care, leading to four fatalities among D-TGA patients (5.2%), whereas 1 D-TGA patients and 2 CC-TGA patients survived.

**Conclusion:**

This study underscores the feasibility of achieving an accurate prenatal diagnosis of TGA during early pregnancy. The utility of prenatal ultrasound in the development of personalized perinatal plans and the application of multidisciplinary treatment during delivery are conducive.

## Introduction

Transposition of the great arteries (TGA) is a common congenital heart condition in which the aorta and pulmonary artery connections are switched (1). TGA account for 5%–7% of patients with congenital heart disease (2). TGA can be categorized into two types. In D-TGA (complete transposition), the atrioventricular connections are preserved, but the ventricular and arterial connections are switched (3). Children with D-TGA are at risk of early pulmonary hypertension and heart failure, often leading to a high mortality rate if left untreated (4). In CC-TGA (corrected transposition), atrioventricular connections are inconsistent, but haemodynamics can be functionally corrected. Children with CC-TGA generally have a more favourable early prognosis. However, long-term complications may arise, such as severe tricuspid regurgitation, atrioventricular block, and right heart failure, often requiring late-stage surgery (5, 6). The outcome varies depending on the presence of associated abnormalities (7, 8).

Advances in prenatal ultrasound diagnostics have significantly improved the detection rate of TGA in fetuses, increasing from 6% in the 1990s to a range of 38% to 68% in recent years (9, 10). Early pregnancy now allows for the detection of TGA (11). Fetal echocardiography offers a means to enhance postnatal risk assessment for TGA (12, 13). This, in turn, enables a comprehensive approach, combining integrated diagnosis with perinatal management tailored to varying risk levels. While there may be relatively high perioperative mortality, the long-term survival of TGA patients is notably positive (14), with 15-year survival rates reaching as high as 90% (15, 16).

Accurate prenatal diagnosis is pivotal for effective prenatal counselling and provides the foundation for early postnatal intervention and treatment. Experts need to issue detailed prenatal reports to facilitate counselling and enable early intervention and treatment. A comprehensive assessment by ultrasound, obstetrics, and paediatric cardiac surgeons during pregnancy is crucial for integrated perinatal management. However, most related studies have focused primarily on obstetrics and paediatric cardiac surgery (17, 18). Fetal echocardiography has the opportunity to perform more accurate postnatal risk stratification for TGA and provide conditions for the combined integrated diagnosis, treatment and management. Our study aimed to explore the relationships between intracardiac and extracardiac malformations, chromosomal abnormalities, the intrauterine course, and postnatal outcomes. We sought to evaluate the value of ultrasonography for diagnosing TGA in fetuses during early pregnancy. Our goal is to improve the prenatal diagnosis and perinatal management of TGA, ultimately leading to improved pregnancy outcomes.

## Materials and methods

### Study population

From January 2012 to September 2022, 121 pregnant women were diagnosed with TGA in the Department of Ultrasound Medicine at Fujian Maternity and Child Health Care Hospital. Of these, 103 pregnant women met the inclusion criteria. The average maternal age was 28.79 ± 4.30 years, and the gestational age ranged from 12 to 39 weeks, with an average of 180.12 ± 25.16 days. All pregnant women and their families were informed about the study and provided consent. The study was approved by the Ethics Committee of Fujian Maternity and Child Health Care Hospital (2018-017). Pregnant women who met criteria (1) and (2) or (3) were included in the study: (1) underwent routine prenatal screening with complete measurements; (2) had pathological examinations, such as cardiac micropathological anatomy during early pregnancy, heart topographic anatomy, or cardiovascular casting during mid-pregnancy, were completed; and (3) had fetal echocardiography, prenatal diagnosis and neonatal congenital heart disease screening performed at our hospital. For women who opted for pregnancy termination, informed consent was obtained to verify the local pathological anatomy or vascular cast.

### Study protocol

The GE Voluson E8 and E10 ultrasonic diagnostic devices were utilized for prenatal assessments. A convex array probe with a frequency range of 2.0–9.0 MHz was used for transabdominal examinations. In patients suspected of having TGA during early pregnancy, an intracavitary probe with a frequency range of 5.0–8.0 MHz was selected. In the neonatal period, the Phillips IPEIQ 7C device was used, and a phased array probe with a frequency ranging from 3.0 to 8.0 MHz was chosen for the examinations. For perinatal management and the collection of follow-up outcomes, relevant data were gathered from medical records.

### Ultrasound examination

Fetal heart exams, including four-section, nine-section, and two-section examinations, were performed during the first, second, and third trimesters, respectively (19, 20). These examinations involved sequential segmental analysis to observe the positions of the atria, ventricles, and great arteries in fetuses with suspected TGA. The primary focus was on assessing the connections between the atria and ventricles, connections between the ventricles and great arteries, the branches and characteristics of the great arteries, the presence of a cross relationship between the great arteries, and the existence of a ventricular septal defect (VSD) and outflow tract stenosis. For fetuses without a VSD, the size and blood flow of the foramen ovale and ductus arteriosus were evaluated. Additionally, the presence of other extracardiac malformations was also observed during these examinations.

### Prenatal screening and diagnosis

Prenatal diagnosis is strongly recommended for all fetuses with TGA. This diagnostic process includes noninvasive methods such as noninvasive prenatal genetic testing (NIPT) for screening fetal chromosomal aneuploidy and mid-trimester serological screening (Down's syndrome screening) for additional genetic assessment. In cases where further evaluation was necessary, amniocentesis and umbilical vein puncture were carried out with informed consent from the pregnant women and their families. Subsequently, G-banded karyotype analysis and single nucleotide polymorphism microarray (SNP array) analysis were performed to evaluate the genetic makeup of the fetuses. These genetic assessments were conducted using fetal tissues or umbilical vein blood, particularly for patients who underwent induced labour.

### Multidisciplinary consultation and verification

For women who opted to continue their pregnancy, our center offered a streamlined process that included assistance in diagnosis and treatment. These steps involved regular ultrasound examinations, the creation of personalized pregnancy plans, collaboration among the obstetrics, ultrasound, and paediatrics departments for neonatal care during delivery, and prompt referral to a paediatric cardiac surgeon when necessary. Live-born infants underwent echocardiography and other imaging examinations at our hospital. Periodic echocardiography is recommended for children undergoing surgery, with follow-up intervals of 6 months and every year after surgery. For all live born children, we followed up by telephone communication. Patients who declined access or could not be reached after three attempts were considered lost to follow-up.

### Statistical analysis

The data analysis was performed using SPSS Statistics 26 software. Descriptive statistics methods were used to summarize and analyse the relevant data. Quantitative data are expressed as the mean ± standard deviation, while qualitative data are presented in terms of frequencies (n) and percentages (%). A significance level of *P* < 0.05 was used to indicate the statistical significance threshold.

## Results

**Figure 1:**
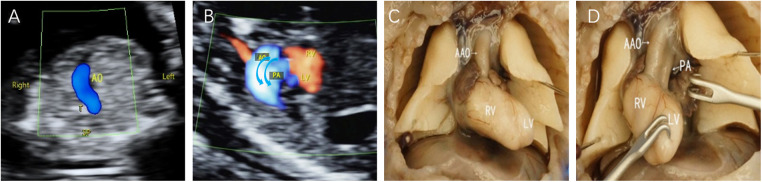
Fetal D-TGA patients were assessed via echocardiography at 13 weeks and 2 days. (**A**) 3VT view showing only one large artery running anteriorly to the right in a “dart-like” pattern. (**B**) Oblique thoracic view showing the juxtaposition of large vessels. (**C,D**) Microscopic dissection of the heart: the two large arteries are aligned in parallel, with the aorta located anteriorly to the right of the right ventricle; the pulmonary artery is located posteriorly to the left of the left ventricle.

**Figure 2:**
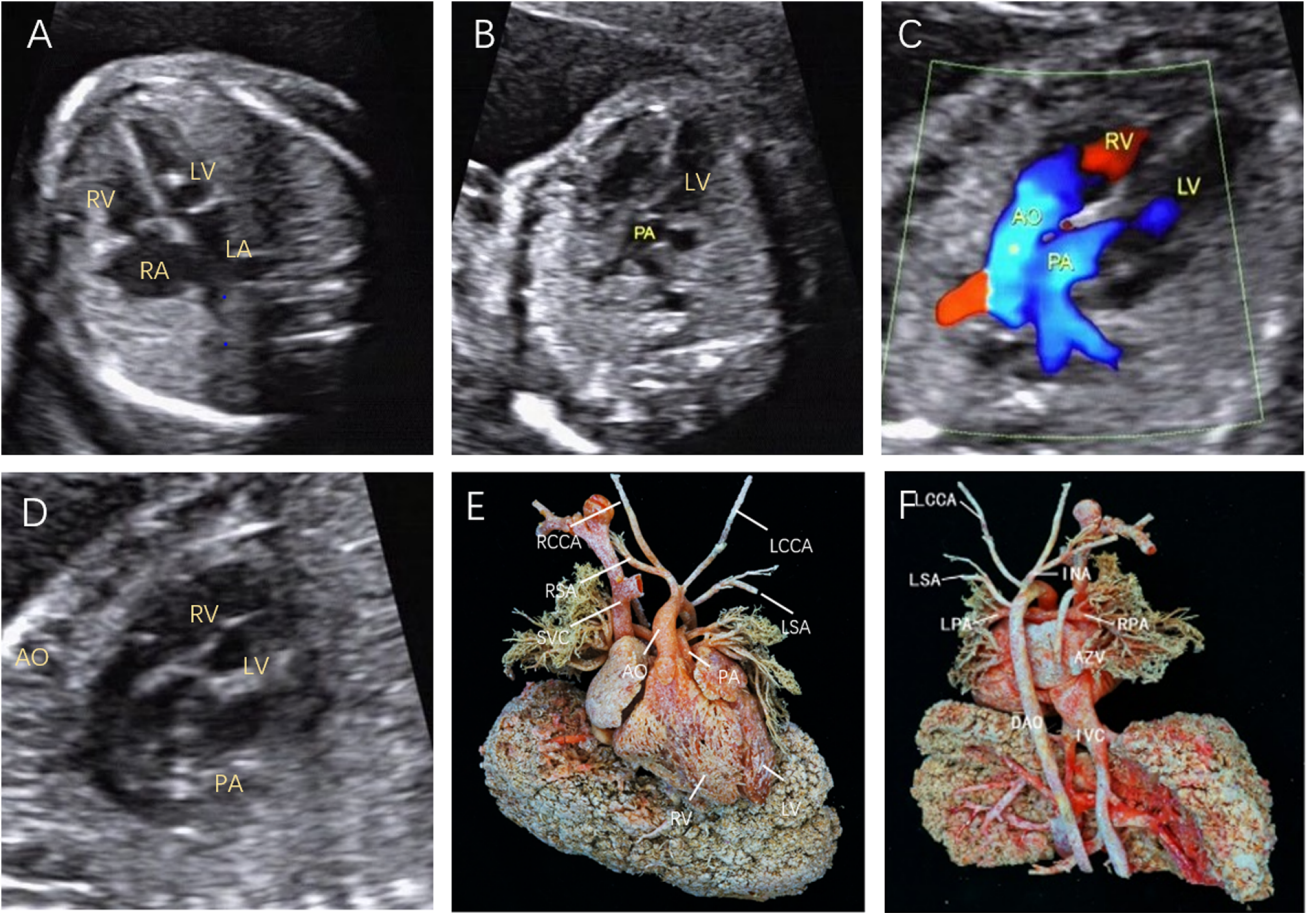
Fetal D-TGA was assessed via echocardiography at 23 weeks and 1 day. (**A**) Four-chamber view with consistent atrioventricular connections and strong dotted echogenicity of the left ventricle. (**B**) Five-chamber view showing the pulmonary artery emanating from the left ventricle and bifurcating into the left and right pulmonary arteries a short distance from the beginning of the artery. (**C**) Two large arteries aligned in parallel, with the aorta emanating from the right ventricle and located anteriorly to the pulmonary artery. (**D**) Long-axis view of the great vessels: the aortic arch emanates from the anterior right ventricle, followed by the head and neck vessels, which curl backwards in a ‘hockey puck’ shape, and the pulmonary artery follows a ‘crutch’ shape in the long-axis view. (**E**) Anatomical cast: frontal view. F: anatomical cast: dorsal view.

**Figure 3:**
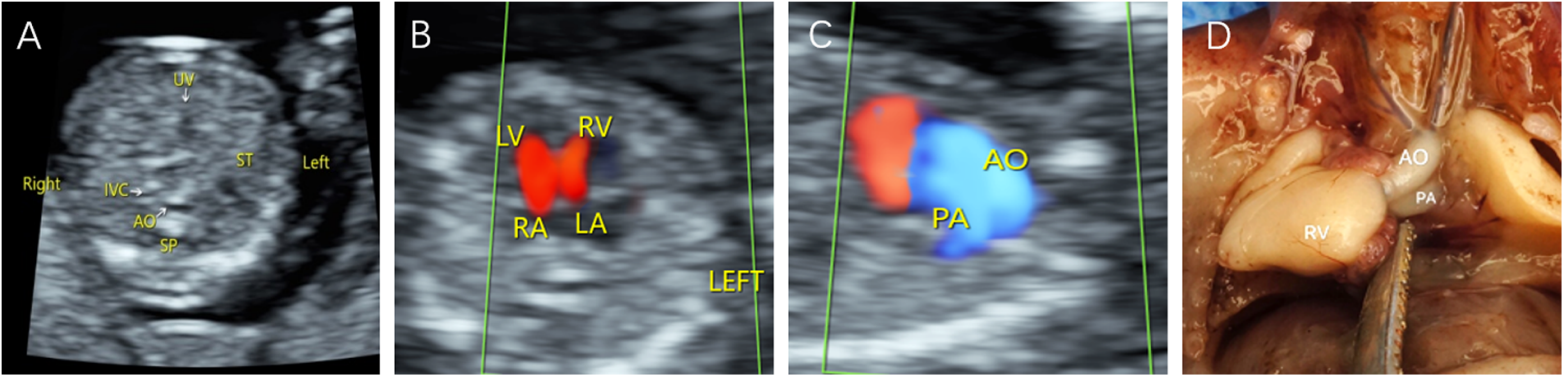
Fetal CC-TGA identified by echocardiography at 12 weeks and 6 days. (**A**) The inferior vena cava and descending aorta are located on the right side of the spine, and the gastric vesicle is on the left side. (**B**) The heart is located in the middle of the thoracic cavity with the apices facing right, and inconsistent atrioventricular connections are observed in the four-chamber view. (**C**) The aorta originates from the right ventricle, the pulmonary artery originates from the left ventricle, and the two great arteries are aligned in parallel. (**D**) Microscopic dissection of the heart: CC-TGA of the right heart.

**Figure 4:**
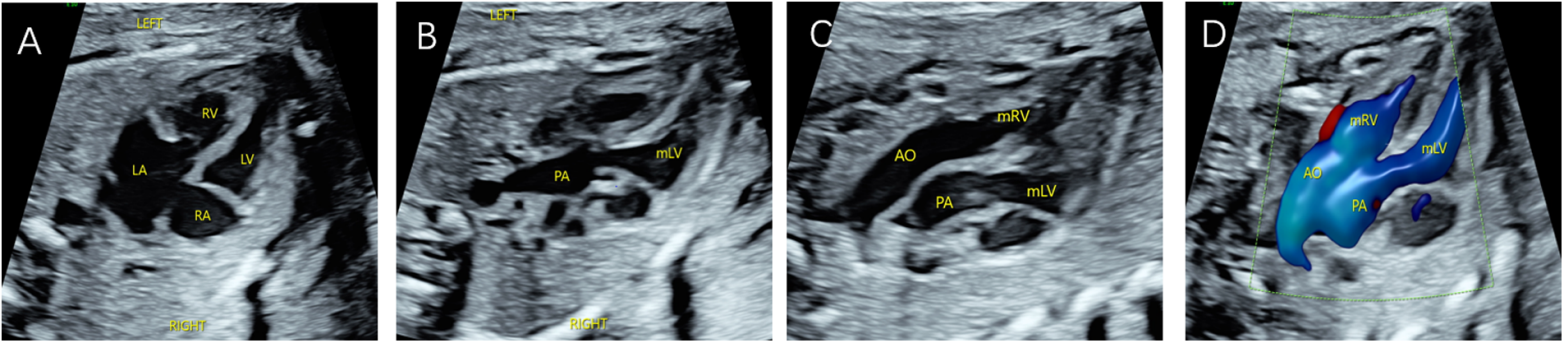
Fetal CC-TGA case confirmed by echocardiography at 23 weeks and 4 days. (**A**) Four-chamber heart view showing inconsistent atrioventricular connections, with the left atrium connected to the morphological right ventricle and the right atrium connected to the morphological left ventricle. (**B–D**) Right ventricular outflow tract view showing the pulmonary artery arising from the morphological left ventricle and left ventricular outflow tract view showing the aorta arising from the morphological right ventricle, with the aorta located anteriorly to the left of the pulmonary artery.

In this study, we assessed 103 fetuses, initially totalling 121 patients with TGA, with 18 patients lost to follow-up. In early pregnancy, D-TGA exhibited characteristic features, including rightward-bent great vessels in the three-vessel-tracheal view, juxtaposed great vessels in the outflow tract oblique view, and consistent atrioventricular connections in the four-chamber view (Figure 1). In the second trimester, the aorta was connected to the right ventricle, and the pulmonary artery was connected to the left ventricle (Figure 2). In early pregnancy, both arteries ran parallel to the aorta anterior to the pulmonary artery congenitally. CC-TGA patients presented with discordant atrioventricular connections (Figure 3). In the second trimester, there were reversed ventricular positions, parallel outflow tracts, pulmonary arteries from the left ventricle, and the aorta from the right ventricle positioned anterior to the pulmonary arteries (Figure 4). The earliest diagnosis occurred at 12 weeks and 3 days of gestation, and 10 mothers (9.7%) were of advanced maternal age. The TGA distribution included 90 D-TGA patients and 13 CC-TGA patients. Diagnoses were made in the first (8 D-TGA, 2 CC-TGA), second (68 D-TGA, 9 CC-TGA), and third trimesters (14 D-TGA, 2 CC-TGA).

### Combined intracardiac and extracardiac abnormalities

We observed that 80 (77.7%) TGA patients presented with intracardiac malformations, while 31 (30.0%) exhibited extracardiac abnormalities. Among the D-TGA patients, 56 (62.2%) had ventricular septal defects (VSDs), 19 (21.1%) had pulmonary artery stenosis, and 9 (10.0%) had tricuspid valve abnormalities. Common extracardiac abnormalities in D-TGA included fetal growth restriction in 11 fetuses (12.2%), thickening of the nuchal translucency/nuchal fold (NT/NF) in 5 fetuses (5.5%), and the presence of a single umbilical artery in 3 fetuses (3.3%). In the case of CC-TGA, the most prevalent intracardiac abnormality was VSD in 8 patients (61.5%), with 5 patients (38.5%) exhibiting pulmonary artery stenosis and 2 patients (15.4%) exhibiting tricuspid valve abnormalities, as detailed in Table 1.

**Table 1:** TGA combined with malformations.

Combined malformations	D-TGA	CC-TGA	Total number
None	21	2	23
Ventricular septal defect	56	8	64
Pulmonary artery stenosis	19	5	24
Tricuspid valve abnormalities	9	2	11
Right aortic arch	6	1	7
Strongly echogenic ventricular foci	5	0	5
Type II atrioventricular block of degree II	0	1	1
Other intracardiac malformations	8	3	11
Fetal growth restriction	11	0	11
NT/NF thickening	5	0	5
Single umbilical artery	3	0	3
Visceral inversion	1	0	1
Fetal nasal bone dysplasia	1	0	1

Other rare combined intracardiac malformations, such as a slightly narrowed aortic arch, right-sided heart, left subclavian artery vagus, and permanent left superior chamber.

### Prenatal screening and diagnosis

Prenatal screening: Among the 103 fetuses diagnosed with transposition of the great arteries (TGA), 46 underwent prenatal screening for Down syndrome. This group included 7 high-risk fetuses, 6 fetuses with D-TGA, and 1 fetus with CC-TGA. Additionally, 16 fetuses underwent NIPT, all of which were classified as low risk. Prenatal Diagnosis: Of the 103 TGA-affected fetuses, 40 underwent prenatal diagnosis. Specifically, 32 patients underwent amniocentesis, while in 8 patients, prenatal diagnosis was carried out using umbilical vein blood samples obtained after labour induction. Chromosomal abnormalities were ascertained in 3 fetuses, encompassing 1 instance of standard trisomy 18, 1 case of partial trisomy 5, and 1 case of 21pstk + polymorphism. Subsequently, all three mothers elected to terminate their pregnancies via labour induction during the second trimester. Comprehensive details are available in Table 2.

**Table 2:** Prenatal screening and prenatal diagnosis in fetal TGA.

	Specimens	Results	D-TGA	CC-TGA	Total
Down's syndrome	Peripheral blood in pregnant women	High risk	6	1	7
	Peripheral blood in pregnant women	Low risk	36	3	39
NIPT	Peripheral blood in pregnant women	Low risk	14	2	16
Chromosome karyotype	Fetal umbilical vein	No significant abnormalities seen	2	0	2
	Amniotic fluid	No significant abnormalities seen	25	3	28
	Amniotic fluid	21pstk + polymorphism.	1	0	1
	Amniotic fluid	Partial trisomy 5	1	0	1
	Amniotic fluid	Standard trisomy 18	1	0	1
Single nucleotide polymorphism microarray	Fetal umbilical vein	No pathogenic genome found	5	1	6
	Amniotic fluid	No pathogenic genome found	19	1	20

NIPT: noninvasive prenatal genetic testing.

### Outcome and prognosis

**Figure 5:**
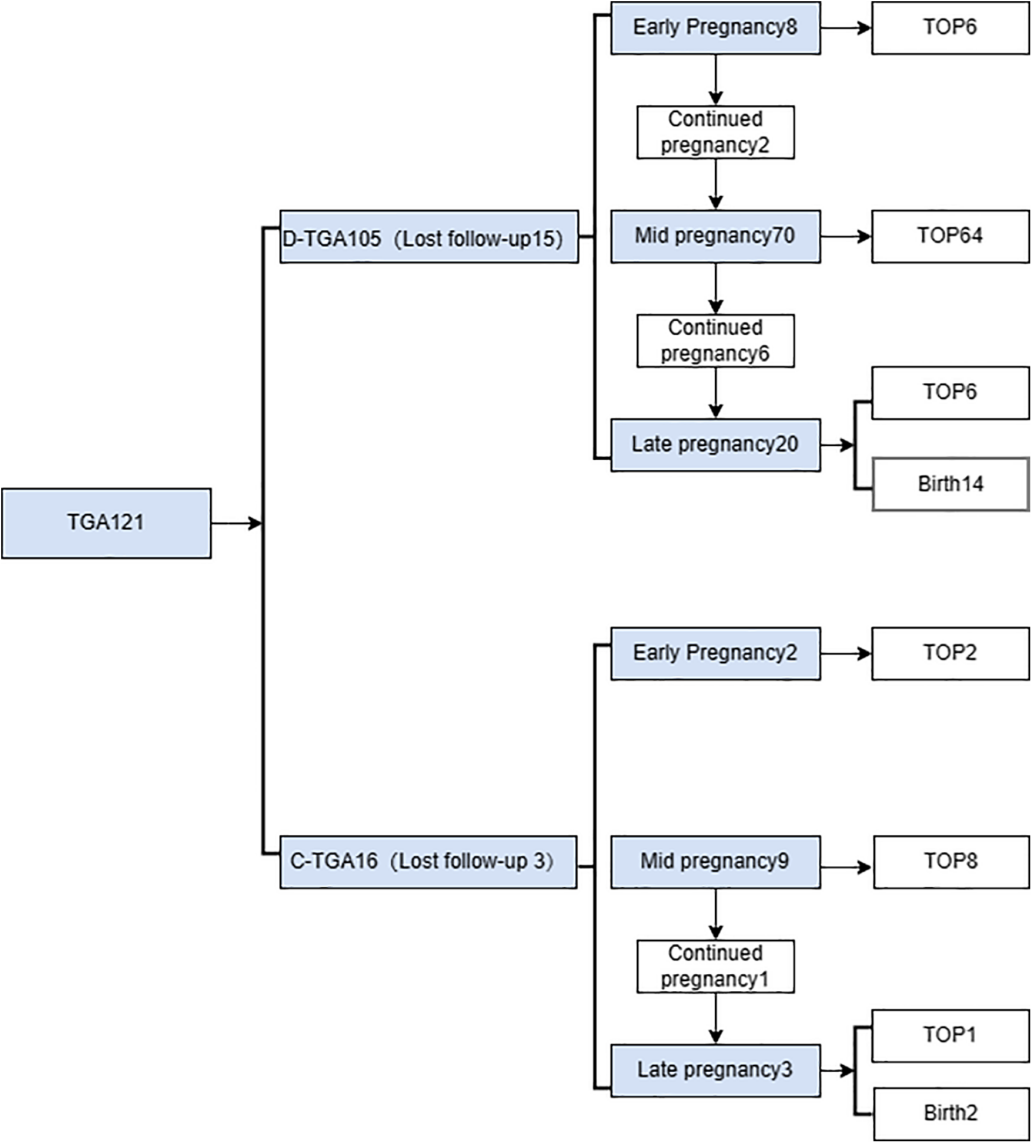
Flow chart of the prenatal diagnosis of transposition of the great arteries from pregnancy.

**Figure 6:**
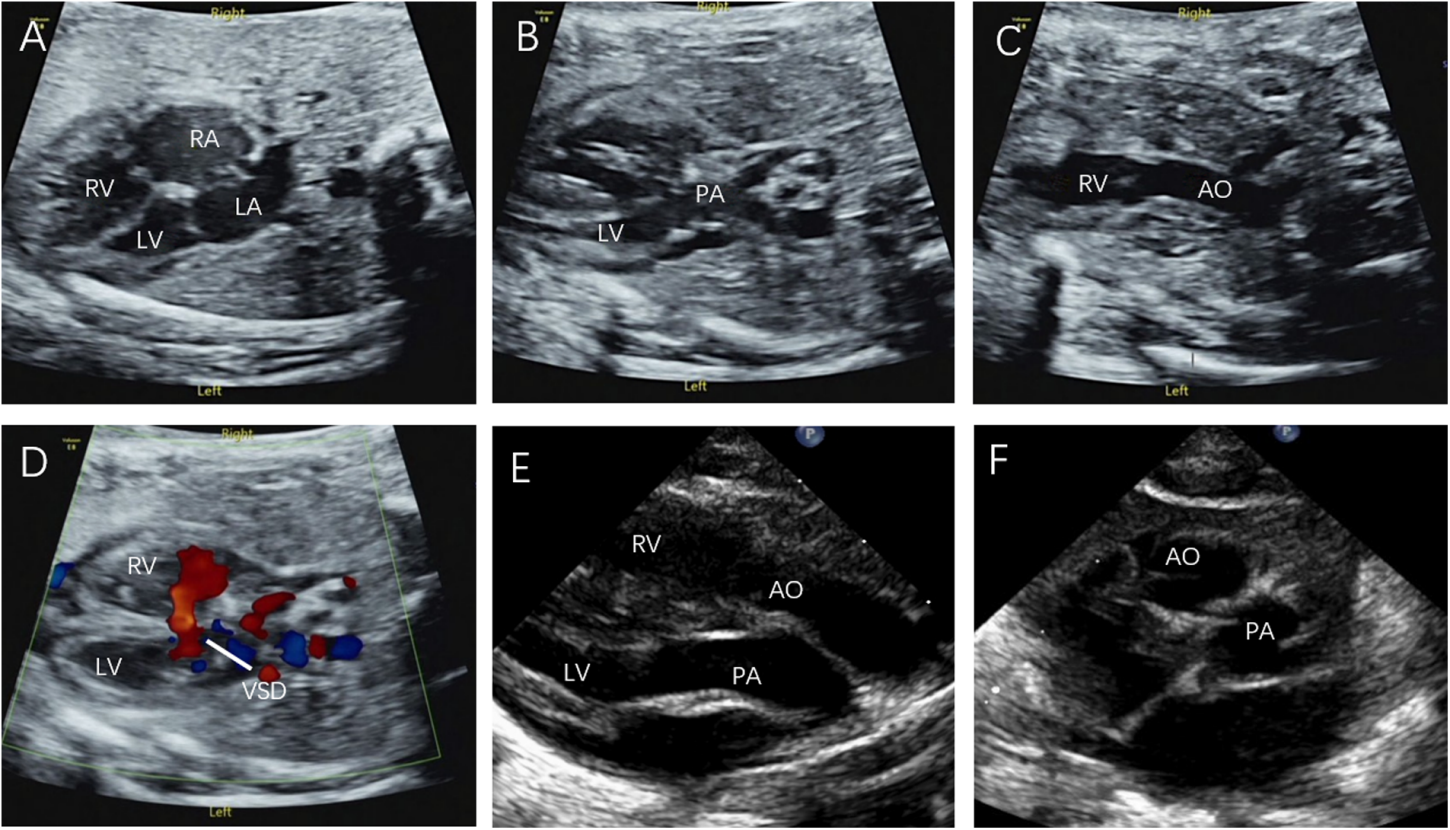
Prenatal and postnatal diagnosis of D-TGA. (**A–D**) D-TGA with VSD at 24 w and 6 d gestation. (**E**) Parasternal long-axis view: The two great arteries are aligned in parallel, with the aorta located anteriorly on the right, originating from the right ventricle, and the pulmonary artery located posteriorly on the left, originating from the left ventricle. (**F**) The short axis view of the great arteries: The aorta and pulmonary artery are circumferential and adjacent rather than travelling normally (the longitudinal pulmonary artery surrounds the circular aorta).

In the present study, 8 D-TGA patients were identified early in pregnancy. Six patients underwent induction of labour (Figures 1C,D), and two were induced in the second trimester. Additionally, 68 D-TGA patients were diagnosed in the second trimester. Of these, 62 underwent induction (Figures 2E,F), and six were observed until delivery. Fourteen patients with late-stage D-TGA were referred to our hospital. Six mothers underwent induction therapy, seven mothers continued their pregnancies at our hospital, and one delivered elsewhere (Figure 5). Fourteen patients experienced continued pregnancies and delivered full-term without preterm issues. Postnatal echocardiography confirmed D-TGA (Figure 6). Among the 9 children (10.0%) who underwent surgery, two passed away postoperatively. The remaining 7 patients achieved positive outcomes. Four children (5.2%) were discharged without surgery due to conditions such as pulmonary inflammation. One (1.3%) patient had no surgery and maintained a positive prognosis by telephone follow-up, as presented in Table 3.

**Table 3:** TGA prenatal ultrasound diagnosis and postnatal live birth.

Number	Combined intracardiac malformations	Consolidation of other cases	Sex	Weeks of termination of pregnancy	Delivery method	Apgar rating	Birth weight (g)	Surgical situation	Prognosis
1	/	/	Male	39w4d	Forceps assisted	9, 9, 9	3,650	Large artery switch	Good
2	/	/	Male	41w6d	Caesarean section	10, 10, 10	3,880	Large artery switch	Good
3	VSD	Excessive amniotic	Female	41w0d	Spontaneous	10, 10, 10	2,700	Large artery switch + VSD repair	Good
4	VSD	/	Male	40w4d	Spontaneous	10, 10, 10	3,270	Large artery switch + VSD repair	Good
5	/	/	Male	38w5d	Spontaneous	10, 9, 9	3,150	Large artery switch	Good
6	VSD	Bilateral renal collecting system separation	Male	38w5d	Caesarean section	10, 10, 10	3,455	Large artery switch + VSD repair	Good
7	/	/	Male	39w2d	Caesarean section	9, 10, 10	3,200	Large artery switch	Good
8	EIF	/	Male		Caesarean section	9, 9, 9	3,520	Large artery switch	Death
9	VSD	/	Male	39w5d	Caesarean section	9, 9, 9	3,700	Large artery switch + VSD repair	Death
10	VSD, Severe TS, PS	/	Male	40w3d	Spontaneous	10, 10, 10	3,350	Without surgery	General
11	PS, VSD, Small PE	/	Female	37w2d	Caesarean section	10, 9, 9	2,890	Palliative care	Death
12	VSD, PS	HC/AC 0.91%、Excessive amniotic	Male	37w1d	Caesarean section	10, 9, 9	3,400	Palliative care	Death
13	VSD	Excessive amniotic	Male	39w6d	Spontaneous	10, 10, 10	2,680	Palliative care	Death
14	RAA	/	Female	37w4d	Spontaneous	10, 10, 10	2,250	Palliative care	Death
15	PS	/	Male	37w3d	Spontaneous	10, 10, 10	3,400	Surgery is not recommended	General
16	VSD, PA, PLSVC	/	Male	38w6d	Spontaneous	10, 10, 10	3,580	Surgery is not recommended	General

Serial numbers 1-14 are D-TGA fetuses; serial numbers 15-16 are CC-TGA fetuses; VSD, ventricular septal defect; EIF, echogenic intracardiac focus; TS, tricuspid stenosis; PE, pericardial effusion; PS, pulmonary artery stenosis; RAA, right aortic arch; PA, pulmonary artery atresia; PLSVC, persistent left superior vena cava.

Two fetuses with CC-TGA were diagnosed in early pregnancy, and both of their mothers chose to induce labour, as presented in Figure 3D. Nine fetuses with CC-TGA were diagnosed in the second trimester; 8 of the fetuses chose to undergo induction of labour, and 1 of the mothers continued pregnancy. Two fetuses with CC-TGA were referred to our hospital in late pregnancy, and after ultrasound confirmation of the diagnosis of CC-TGA in our hospital, the mother of 1 patient chose to induce labour, and the mothers of the other fetuses continued the pregnancy and were delivered at our hospital (Figure 5). Two pregnant women all were monitored until delivery at term without premature delivery. The postnatal echocardiographic findings of fetuses with CC-TGA were consistent with the prenatal findings; surgery was not recommended, and telephone follow-up indicated a good prognosis, as presented in Table 3.

## Discussion

TGA is a prevalent congenital heart condition characterized by cyanosis. In recent years, there has been a consistent increase in the rate of prenatal fetal screening, with the detection rate reaching as high as 77% through comprehensive scanning techniques covering the four-chamber heart section, outflow tract section, and three-vessel trachea section (21, 22). Notably, TGA is typically not linked to chromosomal abnormalities or extracardiac malformations (23). The significance of prenatal diagnosis lies in enhancing management strategies. This approach empowers expectant mothers to make well-informed decisions regarding the continuation of their pregnancies and the formulation of delivery plans. Moreover, this approach facilitates the timely implementation of effective treatments after fetal delivery, thereby reducing the occurrence of severe complications and even fetal or neonatal mortality. In this process, prenatal fetal ultrasound screening plays a pivotal role (24).

Most of the cases in the second trimester were TGA suggested by routine prenatal screening at another hospital and referred to our hospital for further definitive diagnosis. Fifteen cases were missed in our hospital in early pregnancy, which may be because the four-chamber view of TGA color Doppler ultrasound was normal, and the oblique view of the great arteries at the base of the heart was mistaken for three-vessel tracheal view blood flow, which was very similar to the “V” -shaped structure. Even in the second trimester, the four-chamber view of D-TGA cases was almost normal, and the outflow view had some limitations in the face of TGA examination, which only showed the initial segment of the outflow tract, without further showing that the aortic arch or the bifurcation of the main pulmonary artery was the section of the left and right pulmonary arteries, so the connection relationship between the ventricles and the large arteries could not be clarified, and TGA was easily missed. In the diagnosis of TGA, the short axis view of the great arteries is one of the important sections observed, and it is also a common section to observe the anatomical site and classification of ventricular septal defects, which can show the relationship between the two great arteries. The basal outflow tract view more clearly shows the relationship between ventricular septal defects and large arteries (away from and riding span). It helps to identify tetralogy of Fallot, transposition of the great arteries, and double outlet right ventricle.

In this study, 90 fetuses with D-TGA and 13 with CC-TGA exhibited intracardiac malformations. Individualized postnatal treatment, with informed consent, was planned for TGA patients with extracardiac abnormalities. One fetus with D-TGA had rare forebrain and facial dysplasia, prompting a second-trimester induced delivery with consent. Experienced sonographers are recommended to assess cardiac and extracardiac abnormalities for prenatal TGA screening, facilitating multidisciplinary care. Chromosomal abnormalities have rarely been reported in D-TGA patients (25, 26). Prenatal diagnosis, with informed consent, is advisable to exclude these. Moreover, 3 out of 40 D-TGA fetuses (7.5%) had chromosomal abnormalities, which was greater than that in the general population. Only one of the three fetuses had diaphragmatic hernia, and the rest had no other extracardiac abnormalities. CC-TGA patients had a lower incidence of disease (27), with no significant abnormalities detected in the four examined patients. D-TGA has a minimal impact on fetal development due to the open foramen ovale and ductus arteriosus. However, immediate intervention is crucial after birth to prevent severe cyanosis and hypoxemia, which are typically achieved through balloon atrial septostomy (28). An arterial switch operation within the first few weeks of life for newborns with D-TGA leads to a better prognosis (29). Prostaglandin infusion and balloon atrial septostomy are often required for isolated patients to improve oxygenation (14, 28). In congenital CC-TGA patients, where haemodynamics are functionally corrected, the prognosis is generally good (30). However, the outcomes depend on associated cardiac defects, and studies have focused on combining these malformations (7, 31). Early prenatal diagnosis allows for informed consultations and timely interdisciplinary care postdelivery.

In this study, 77 (74.8%) pregnant women were diagnosed in the second trimester and chose to terminate pregnancy 72 (69.9%), possibly because systematic prenatal ultrasonography was routinely performed in the second trimester in China, which included screening for cardiac malformations. Currently, the gestational age at diagnosis of TGA ranges from 12 to 39 weeks, with the earliest diagnosis occurring at 12 weeks and 3 days. Because the fetal heart volume is small in early pregnancy, it is difficult to make a diagnosis of transposition of the great arteries, and for those suspected of TGA in the first trimester, because it is difficult to make a comprehensive evaluation of cardiac structure and function at this time, in addition to the lack of clear gestational age requirements for termination of pregnancy in China, it is often recommended to reexamine after 18 weeks of gestation. Only eight D-TGA patients and two CC-TGA patients were identified early in pregnancy, leading to recommendations for further genetic examination. Ten fetuses with TGA underwent Down syndrome screening, all of which indicated low risk. Pregnant women were given the choice to continue or terminate the pregnancy. Six cycles of D-TGA and two cycles of CC-TGA were terminated. Specimens from terminated pregnancies were microdissected for verification. For those who continued their pregnancies, the center provided comprehensive support, including regular ultrasound examinations and detailed measurements and observations following fetal echocardiography guidelines. The goal was to ensure accurate and complete diagnosis. In one patient, a D-TGA patient with VSD was diagnosed early and delivered after induced labour during the second trimester. In another D-TGA patient with VSD, dextrocardia, preaxial polydactyly of both hands, and situs invs. were terminated by choice.

Unless in presence of severe extra-cardiac anomalies or diseases associated to TGA, termination is not recommended as the long term outcomes of both operated TGA and operated or not operated CC-TGA are well known (14, 31). In addition, the perinatal management of TGA are extremely well established in order to achieve adequate cardiovascular stability of the newborn. The high induced labour rate of TGA fetuses in our center may be due to our fertility policy. Originally, China advocated that a couple give birth to only one child. Most families wanted their only child to be completely healthy; therefore, if fetal heart abnormalities were found, induction of labour would be selected. However, currently, the three-child policy has been implemented (32), and society has begun to regard fetuses as complete lives and identify fetuses with abnormal hearts as patients with fetal heart disease. With the development of medical technology, many congenital heart diseases are now good treatments, induction of labour is no longer the only solution for congenital heart disease, and the future induction of the labour rate will decrease.

Ultrasound aims to provide scientific and objective medical information for integrated prenatal and postnatal management. When TGA is diagnosed by prenatal and preoperative ultrasound, special attention should be paid to: (1) the continuity of the interventricular septum, intact or only combined with small ventricular septal defect in children are more critical and require early treatment in the neonatal period; (2) aortic valve and pulmonary valve morphology, leaflet number, leaflet opening and closing function; (3) the presence or absence of stenosis under the pulmonary valve, left pulmonary artery and right pulmonary artery development. Postoperative ultrasound should focus on: (1) aortic conditions, almost all cases are associated with aortic root dilatation, resulting in different degrees of aortic regurgitation, the incidence of ascending aortic stenosis is about 5% (33, 34); (2) pulmonary artery conditions, about 10% of patients with progressive supravalvular pulmonary stenosis, pulmonary artery branch stenosis is also one of the common complications (14, 35); (3) VSD with aortic coarctation in children can also be well corrected, but long-term prognosis analysis showed that the risk of arch re-constriction and right ventricular outflow tract obstruction is high (33); (4) postoperative arrhythmia accounts for about 10% (34).

The direction of prenatal diagnosis should align with that of integrated management, including improving the secondary and tertiary prevention systems for birth defects and establishing a multidisciplinary diagnosis and treatment consultation platform to provide effective individualized evaluation and treatment for fetuses with cardiovascular malformations. This approach ensures timely and effective treatment through a streamlined process.

### Limitations

This study has several limitations, as it included a small number of patients from a single center, a retrospective study design, a limited number of pregnant women undergoing karyotyping or SNP array examination, and a relatively short follow-up duration. The lesions of the fetal brain and nervous system have not been reported, which would have great clinical significance. Moreover, further research is needed to investigate the mobility, mortality, and quality of life of TGA patients to improve prenatal counselling. Later follow-up should be 1 year, 3 years, 5 years, and cover the heart, development and nervous system status, will be more perfect.

## Conclusion

Precise TGA diagnosis can be achieved in early pregnancy through echocardiography. For expectant mothers opting to proceed with their pregnancies, comprehensive prenatal ultrasound monitoring, personalized perinatal management, and multidisciplinary intervention during delivery all play pivotal roles in optimizing outcomes within the TGA population.

## Data Availability

The original contributions presented in the study are included in the article/Supplementary Material, further inquiries can be directed to the corresponding authors.
